# ParB deficiency in *Pseudomonas aeruginosa* destabilizes the partner protein ParA and affects a variety of physiological parameters

**DOI:** 10.1099/mic.0.024661-0

**Published:** 2009-04

**Authors:** A. A. Bartosik, J. Mierzejewska, C. M. Thomas, G. Jagura-Burdzy

**Affiliations:** 1 Institute of Biochemistry and Biophysics, PAS, 02-106 Warsaw, Pawinskiego 5A, Poland; 2 School of Biosciences, University of Birmingham, Edgbaston, Birmingham B15 2TT, UK

## Abstract

Deletions leading to complete or partial removal of ParB were introduced into the *Pseudomonas aeruginosa* chromosome. Fluorescence microscopy of fixed cells showed that ParB mutants lacking the C-terminal domain or HTH motif formed multiple, less intense foci scattered irregularly, in contrast to the one to four ParB foci per cell symmetrically distributed in wild-type *P. aeruginosa*. All *parB* mutations affected both bacterial growth and swarming and swimming motilities, and increased the production of anucleate cells. Similar effects were observed after inactivation of *parA* of *P. aeruginosa*. As complete loss of ParA destabilized its partner ParB it was unclear deficiency of which protein is responsible for the mutant phenotypes. Analysis of four *parB* mutants showed that complete loss of ParB destabilized ParA whereas three mutants that retained the N-terminal 90 aa of ParB did not. As all four *parB* mutants demonstrate the same defects it can be concluded that either ParB, or ParA and ParB in combination, plays an important role in nucleoid distribution, growth and motility in *P. aeruginosa*.

## INTRODUCTION

Representatives of two families of bacterial Par proteins, ParA and ParB, are encoded by the majority of bacterial chromosomes in the close vicinity of *oriC* (Gal-Mor *et al.*, 1998; Ogasawara & Yoshikawa, 1992); the exceptions are *Enterobacteriaceae* and *Pasteurellaceae*, which appear to lack these loci. The first Par proteins were studied in low-copy-number plasmids, where they were shown to drive active partitioning of plasmid molecules to the progeny cells (Bignell & Thomas, 2001).

Increasing data from different *par* systems have established that Par proteins interact with each other to play their role in plasmid or chromosome partitioning (Autret *et al.*, 2001; Bartosik *et al.*, 2004; Ebersbach & Gerdes, 2004; Erdmann *et al.*, 1999; Figge *et al.*, 2003; Kim & Shim, 1999; Lukaszewicz *et al.*, 2002; Ravin *et al.*, 2003). Component A of the partitioning system is an ATPase either of Walker-type (ParA) or actin-like (ParM) and forms dynamic structures within bacterial cells (Adachi *et al.*, 2005; Davey & Funnell, 1994; Davis *et al.*, 1992; Ebersbach & Gerdes, 2004; Easter & Gober, 2002; Jensen & Gerdes, 1997; Marston & Errington, 1999; Quisel & Grossman, 2000). Component B is a DNA-binding protein that recognizes the centromere-like sequence(s), usually called *parS*, and forms regularly distributed foci (in complex with DNA) in bacterial cells irrespective of whether it is encoded on the chromosome or a plasmid (Biek & Strings, 1995; Bignell *et al.*, 1999; Bouet *et al.*, 2000; Erdmann *et al.*, 1999; Funnell, 1991; Glaser *et al.*, 1997; Gordon *et al.*, 1997; Lewis & Errington, 1997; Niki & Hiraga, 1997). The nucleoprotein complexes formed by the components of the *par* systems are required for accurate partitioning of plasmids prior to cell division.

Studies on chromosomally encoded Par proteins in *Bacillus subtilis*, *Caulobacter crescentus*, *Streptomyces coelicolor*, *Pseudomonas putida*, *P. aeruginosa* and, recently, *Helicobacter pylori*, *Burkholderia cenocepacia*, *Vibrio cholerae* and *Mycobacterium smegmatis* have indicated their involvement (to different extents in different species) in chromosome partitioning but also in other cell processes (Bartosik *et al.*, 2004; Dubarry *et al.*, 2006; Godfrin-Estevenon *et al.*, 2002; Ireton *et al.*, 1994; Jakimowicz *et al.*, 2007a, b; Kim *et al.*, 2000; Lee *et al.*, 2006; Lewis *et al.*, 2002; Lin & Grossman, 1998; Mohl & Gober, 1997; Saint-Dic *et al.*, 2006).

In most chromosomal *par* systems studied to date mutations in the *par* genes can be easily generated, indicating that these genes are not essential for growth. The exception so far is *C. crescentus*, in which Par proteins are involved in the regulation of cytokinesis and gene knockouts are lethal (Figge *et al.*, 2003; Mohl *et al.*, 2001). In *B. subtilis* the ParA homologue Soj was first described as the regulator of the entry into sporulation. The ParB homologue Spo0J is required for both initiation of sporulation and normal chromosome partitioning during vegetative growth. Mutations in *spo0J* lead to an increased number of anucleate cells, defects in separation of replicated origins and impaired regulation of replication initiation during the vegetative cell cycle (Ireton *et al.*, 1994; Lee *et al.*, 2003; Lee & Grossman, 2006). Mutations in *soj* (*parA*) have no significant effect on chromosome segregation in *B. subtilis* although the chromosome becomes decondensed. A *soj spo0J* double mutant is impaired in chromosome partitioning during sporulation (Sharpe & Errington, 1996). For *P. putida parA*, *parB* or double *parAB* mutants grown in minimal medium 5–10 % of cells were anucleate during the transition from exponential growth to stationary phase and it was suggested that the Par proteins in this species are particularly important for chromosome partitioning when the bacteria are undergoing cell division in the absence of ongoing DNA replication (Lewis *et al.*, 2002; Godfrin-Estevenon *et al.*, 2002).

**Fig. 1. f1:**
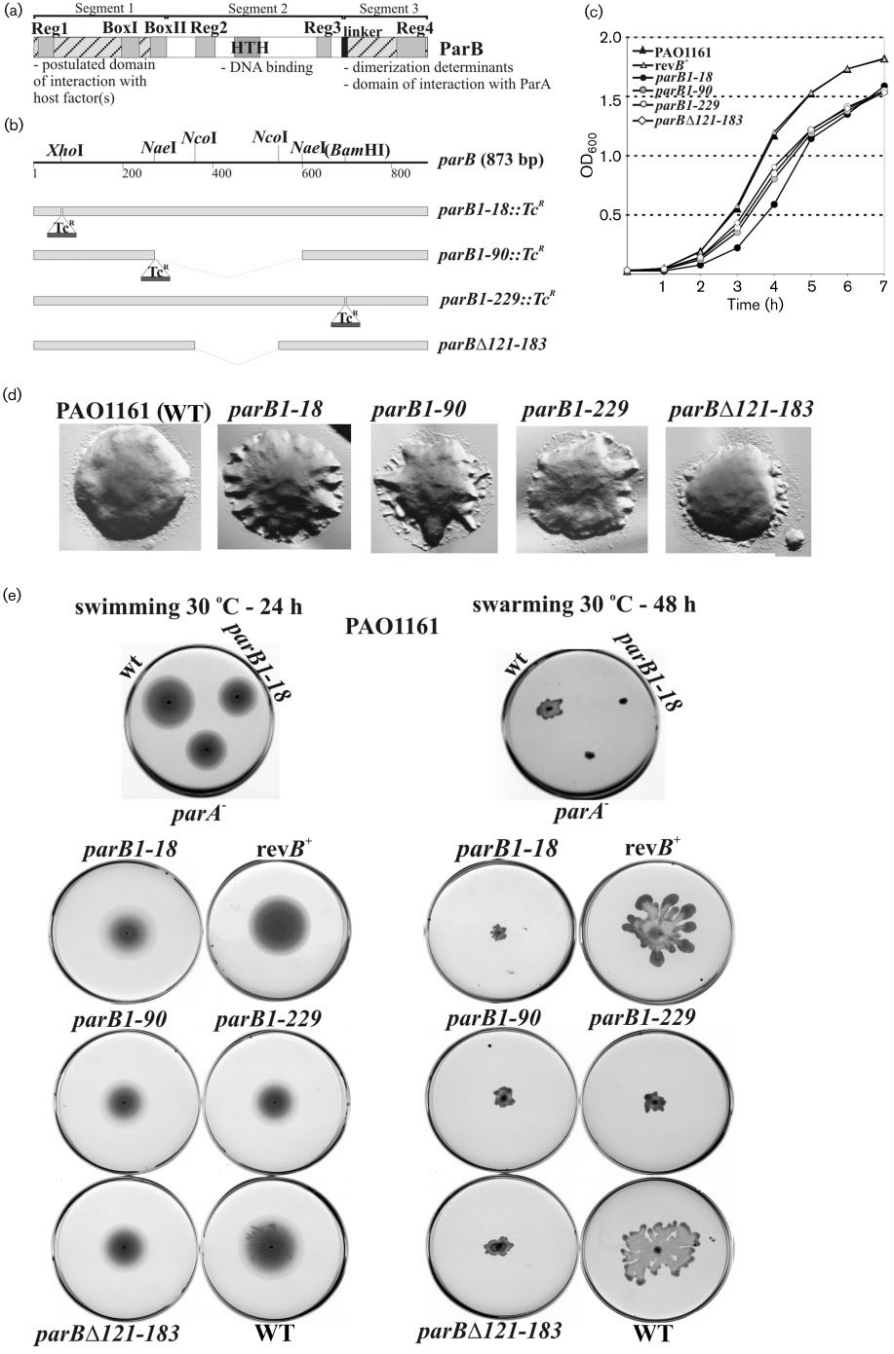
Effect of *parB* mutations on growth, colony formation, and swimming and swarming motility of *P. aeruginosa*. (a) Domain structure of ParB of *P. aeruginosa*. The conserved regions are marked in grey. The linker region is marked in black. The N-terminal domain of interaction with putative host factors and C-terminal domain involved in dimerization are hatched. (b) The *parB* gene from *P. aeruginosa* and its mutant derivatives constructed for allele replacement. The restriction sites shown are those used in manipulations. The tetracycline-resistance cassette (Tc^R^; not to scale) comes from plasmid pKRP12 (Reece & Phillips, 1995). (c) Growth of WT PAO1161 strain, *parB* mutants and *parB^+^* revertant strain (rev*B*^+^) on rich medium at 37 °C. Overnight cultures were diluted 100-fold. The OD_600_ was measured at hourly intervals. (d) Colony morphology of WT and *parB* mutant strains observed after 24 h incubation on L agar plates at 37 °C using an Olympus IX70 microscope. Photomicrographs were projected, and visualized with DP-Soft (analySIS) software produced by Soft Imaging Systems for Olympus. The final montage was created with Adobe Photoshop, version 6.0. (e) Motility of WT, *parB* mutants and *parB^+^* revertant strain tested as described in Methods. The test plates were inoculated with a sterile toothpick and incubated for 24–48 h at 30 °C. The *parA^−^* strain was included to demonstrate the similarity to the ParB^−^ phenotype.

In the sequenced *P. aeruginosa* PAO1 genome the *parA* and *parB* genes were identified in a region about 8 kb from *oriC*. As in many other eubacterial chromosomes the *P. aeruginosa par* genes are situated close to genes implicated in DNA replication, cell division and basic metabolism. Comparative sequence analysis of ParB representatives uncovered conserved motifs within the protein, regions 1 to 4 (Fig. 1a[Fig f1]) (Bartosik *et al.*, 2004), in addition to previously identified sequences of unknown function, ParB Box I and Box II (Yamaichi & Niki, 2000). The HTH motif (the putative domain of interaction with DNA) is located in the central part of ParB protein and the dimerization determinants are located in the C terminus (Bartosik *et al.*, 2004). Ten putative *parS* sites were identified in the *P. aeruginosa* chromosome and exhibited a hierarchy of binding strengths for ParB*_P.a._ in vitro* (Bartosik *et al.*, 2004). *In vivo* experiments demonstrated that ParB*_P.a._* is able to bind to palindromic *parS_2/3_* and silence the expression of genes in close proximity to the binding site. ParA, ParB and *parS_2/3_* of *P. aeruginosa* can act as a plasmid active partitioning system (Bartosik *et al.*, 2004). The overproduction of ParA (Lasocki *et al.*, 2007) or ParB (Bartosik *et al.*, 2004) disturbs cell division and nucleoid distribution. The ‘toxicity’ determinants (causing growth retardation when overproduced) have been mapped to the N-terminal 85 aa of ParA (Lasocki *et al.*, 2007) and N-terminal 90 aa of ParB (Bartosik *et al.*, 2004). The inactivation of chromosomal *parA* is not lethal but leads to a more than 100-fold increase in the number of anucleate cells even in actively dividing cultures. The growth rate is reduced and cells are impaired in two types of motility: drastically in swarming and to a lesser extent in swimming. The observed defects cannot be complemented by *parA in trans*, suggesting that the amount of Par proteins as well as the ParA : ParB ratio is very important. The ParB produced by *parA* null mutants is prone to degradation so it was difficult to separate which effects of *parA* mutation are due to the lack of ParA and which to a decreased level of ParB in the cells (Lasocki *et al.*, 2007). In this study, to distinguish between these effects and to better determine the role of ParB in the *P. aeruginosa* cell cycle, strains completely or partially deleted for *parB* were constructed. We found that mutations in *parB* are not lethal for *P. aeruginosa* and have pleiotropic effects on chromosome segregation, colony formation and motility similar to the phenotype of *parA* mutants (Lasocki *et al.*, 2007). Complete deletion of *parB* resulted in instability of ParA, but as three of the four *parB* mutants do not affect ParA level we can conclude that impairment or loss of ParB is a key reason for the observed defects in both *parB* and *parA* mutants.

## METHODS

### Bacterial strains and growth.

*Escherichia coli* strains used were K-12 strain DH5*α* [F^−^ (*φ80*d*lacZ*ΔM15) *recA1 endA1 gyrA96 thi-1 hsdR17* (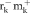) *supE44 relA1 deoR* Δ(*lacZYA–argF*)*U196*], B strain BL21 [F^−^ *ompT hsdS*_B_ (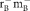) *gal dcm* (*λ* DE3)] (Novagen) and S17-1 (*pro hsdR hsdM recA* Tp^R^ Sm^R^ ΩRP4-Tc : : Mu-Km : : Tn*7*) (Simon *et al.*, 1986). *Pseudomonas aeruginosa* PAO1161 (*leu* r^−^m^+^) was kindly provided by B. M. Holloway (Monash University, Clayton, Victoria, Australia). PAO1161 Rif^R^ was isolated during growth with 125 μg rifampicin ml^−1^. Bacteria were grown in L broth (Kahn *et al.*, 1979) at 37 °C or 30 °C or on L agar (L broth with 1.5 %, w/v, agar) supplemented with antibiotics as appropriate: benzylpenicillin sodium salt at 150 μg ml^−1^ in liquid medium and 300 μg ml^−1^ on agar plates for penicillin resistance in *E. coli*, streptomycin sulphate at 30 μg ml^−1^ for streptomycin resistance in *E. coli* and 200 μg ml^−1^ in *P. aeruginosa*, tetracycline at 10 μg ml^−1^ for tetracycline resistance in *E. coli* and 100 μg ml^−1^ for *P. aeruginosa*, carbenicillin at 300 μg ml^−1^ for carbenicillin resistance in *P. aeruginosa*, rifampicin at 300 μg ml^−1^ for rifampicin resistance in *P. aeruginosa*. Some experiments were performed in M9 minimal medium (Sambrook *et al.*, 1989). The L agar used for blue/white screening contained 0.1 mM IPTG and X-Gal at 40 μg ml^−1^.

### Plasmid DNA isolation, analysis, cloning and manipulation of DNA.

Plasmid DNA was isolated and analysed by standard procedures (Sambrook *et al.*, 1989). The plasmids used in this study are listed in Table 1. Details of the cloning procedures are available as supplementary data with the online version of this paper (Supplementary Methods).

Standard PCR (Mullis *et al.*, 1986) was performed as described previously (Lasocki *et al.*, 2007) with the following pairs of primers for *parB* or *gidBparAparB* amplification: ParB1 (5′-CCGAATTC**ATG**GCAGCCAAGAAACGTGG-3′) and ParB2 (5′-CCGTCGACCCGACTACCCGCTACAACCC-3′); ParB1 and ParB3 (5′-GGGGATCCGTCCACTGCCGAACCAGGGC-3′); ParB4 (5′-GGGGATCCCCTGCGGGACCGGTCAAGAG-3′) and ParB2; GidB1 (5′-CCAAGCTT**ATG**TCTGCGGTAACCCA-3′) and ParB2 (restriction enzyme recognition sites underlined; start codons in bold). Chromosomal DNA of *P. aeruginosa* PAO1161 or PAO1161*parB* mutant strains was used as the template in appropriate PCRs.

DNA sequencing was performed at an internal sequencing facility (IBB PAS, Warsaw, Poland) by using the dye terminator method in conjunction with an ABI 373 automated DNA sequencer.

### Bacterial transformation.

Competent cells of *E. coli* were prepared by the standard CaCl_2_ method (Sambrook *et al.*, 1989). Competent cells of *P. aeruginosa* were prepared by the method of Irani & Rowe (1997).

### Introduction of *par* mutant alleles into *P. aeruginosa* PAO1161.

*E. coli* S17-1 was transformed with pAKE600 suicide vector derivatives (El-Sayed *et al.*, 2001). Transformants were conjugated with the recipient strain *P. aeruginosa* PAO1161Rif^R^ in which the suicide vector with a pMBI *ori* was unable to replicate (see Supplementary Methods). To obtain the PAO1161*parB^+^* revertant strain an *E. coli* S17-1(pABB611) transformant was conjugated with the recipient strain PAO1161*parB1–18* : : *Tc^R^*. Reversion of *parB1–18* : : *Tc^R^* mutation to wild-type *parB* was confirmed by PCR, sequencing and Western blot analysis.

### Protein analysis.

The growth of bacteria was monitored by measuring the OD_600_; the cultures were diluted and plated on L agar to determine c.f.u. ml^−1^. Bacteria were harvested, resuspended in sonication buffer (50 mM phosphate buffer pH 8.0 and 300 mM NaCl) and disrupted by sonication. Cleared extracts after centrifugation were analysed by SDS-PAGE followed by Western blotting performed as described previously (Bartosik *et al.*, 2004).

### Motility assays.

The swimming and swarming assays were performed according to Rashid & Kornberg (2000) with modifications described previously (Lasocki *et al.*, 2007).

### DAPI staining and immunofluorescence microscopy.

The DAPI staining procedure and immunofluorescence microscopy (using FITC-conjugated secondary antibodies) was carried out as previously described (Bartosik *et al.*, 2004; Bignell *et al.*, 1999). Cells were studied using an Olympus IX70 inverted reflected-light fluorescence microscope fitted with a Sensys CCD camera (Photometrics). Images were captured and manipulated on a Macintosh G3 with the Smartcapture I program (Digital Scientific).

### Fluorescence microscopy of living cells.

PAO1161(pABB101) or (pABB131) transformants were grown in selective L broth medium at 24 °C to facilitate observations of the thermolabile EGFP. When cultures reached an OD_600_ of about 0.2, IPTG (0.2 mM) was added to induce the expression of the fusion proteins. After 2–3 h induction (OD_600_ 0.5–0.9), samples were collected and immediately used to prepare microscope slides. Two microlitres of 1.5 % low-melting agarose (Bio-Rad) solution in L broth was put onto a 76×25×1 mm objective slide. The coated slide was left to solidify and dry in the open air at room temperature for about 10 min. A 5 μl drop of the cell suspension was placed on the surface of the agarose and immediately covered with a coverslip and studied as described above.

## RESULTS

### Mutations in *parB* result in the combination of growth, chromosome segregation and motility defects

ParB proteins appear to consist of three segments: an N-terminal segment 1 (containing conserved region 1, Box I and Box II) of as yet ill-defined function, an internal segment 2 with the DNA-recognition domain (containing the HTH motif, and conserved regions 2 and 3) and C-terminal segment 3 with the dimerization domain (highly conserved region 4) (Fig. 1a[Fig f1]; Bartosik *et al.*, 2004). Segments 2 and 3 are joined by a variable linker. To investigate *P. aeruginosa* ParB function, the *parB* gene was cloned in *E. coli* and mutant alleles were constructed (Fig. 1b[Fig f1]) that produced only the first 18 aa of ParB (*parB1–18* : : *Tc^R^*), or ParB that had lost segments 2 and 3 (*parB1–90* : : *Tc^R^*) or only segment 3 (*parB1–229* : : *Tc^R^*) or part of segment 2 (*parB*Δ*121–183*). The latter two mutant proteins were overexpressed in *E. coli* after cloning into pET28mod and purified with a His_6_-tag at the N terminus. DNA-binding activity of both proteins and their oligomeric state were determined *in vitro* by electrophoretic mobility shift assays and glutaraldehyde cross-linking using wild-type (WT) ParB as the control. Loss of the C terminus in ParB1–229 resulted in a monomeric (as shown previously: Bartosik *et al.*, 2004), sequence-specific DNA-binding protein with >5-fold decreased affinity for *parS* (Supplementary Fig. S1A) whereas deletion of segment 2 (*parB*Δ*121–183*) resulted in loss of the ability to bind to DNA (Supplementary Fig. S1A) but retention of the dimeric state typical for WT ParB as expected (Supplementary Fig. S1B).

The mutated alleles of *parB* were then introduced into the *P. aeruginosa* PAO1161 chromosome by a two-step allelic exchange as previously described (El-Sayed *et al.*, 2001). The presence of the mutations was confirmed by PCR analysis and the linked selectable *tetA* marker where applicable. Western blotting with anti-ParB antibodies detected the truncated proteins ParB1–229 and ParBΔ121–183 in the extracts of the respective mutant strains at twofold lower level than observed for WT ParB, whereas the shorter ParB products (in *parB1–18* and *parB1–90* strains) could not be detected at all (data not shown). At no stage of mutant construction was there great difficulty in achieving success, suggesting that the mutations do not have a major effect on viability.

Growth rate (as mean growth rate) of all the *parB* mutants was 5–10 % lower than PAO1161 in both L broth (Fig. 1c[Fig f1]) and minimal medium (data not shown). Cell size of the *parB* mutants increased proportionately (2.33 μm for mutant cells versus 2.20 μm for PAO1161 in L broth and 1.86 μm versus 1.60 μm in minimal medium); the corresponding values in stationary phase were 1.88 μm for mutant cells versus 1.81 μm for PAO1161 in L broth and 1.65 μm versus 1.49 μm in minimal medium, respectively (on samples of at least 2000 cells with standard deviation 0.05 μm).

The colony morphology of all *parB* mutants was irregular, with lobed or jagged edges as compared to the smooth-edged circular colonies of the WT (Fig. 1d[Fig f1]). All four *parB* mutants, despite having either the HTH motif or/and the dimerization domain deleted, exhibited defects in swarming and swimming (Fig. 1e[Fig f1]) but not in twitching (data not shown). Similar phenotypes were observed previously for a *parA* mutant (Lasocki *et al.*, 2007).

Epifluorescence microscopy after staining DNA with DAPI (Supplementary Fig. S2a) showed that *parB* mutants demonstrated a >100-fold increase in the number of anucleate cells during growth on rich medium: from 0.02 % for WT to about 2 % in mutant cultures among actively growing cells and to 4 % in stationary-phase cultures (on samples of at least 1000 cells). The mutant strains grown on minimal medium contained up to 4 % or 7 % of chromosome-less cells in the exponential and stationary phase of growth, respectively (on samples of at least 1000 cells). In addition about 5 % of the cells appeared to be undergoing division without separation of chromosomes (Supplementary Fig. S2A).

### ParB is necessary for ParA stability in *P. aeruginosa*

**Fig. 2. f2:**
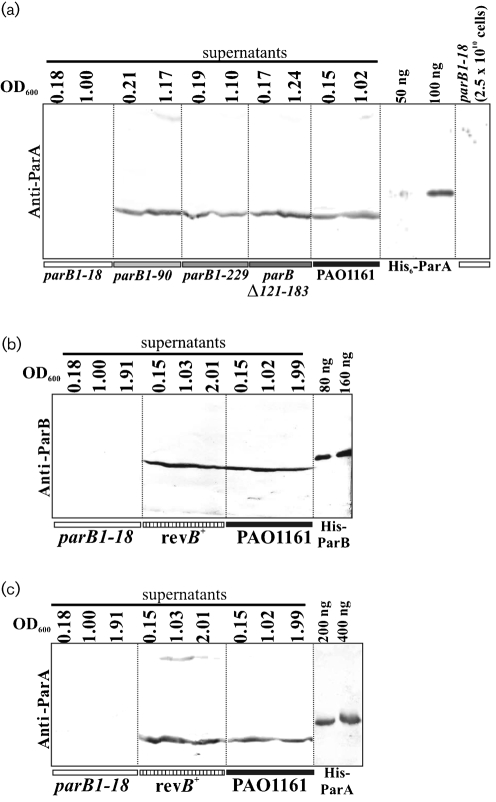
Analysis of ParA and ParB levels in WT and mutants of *P. aeruginosa*. (a) Samples from early- and late-exponential-phase cultures at the indicated OD_600_ were collected and sonicated. The cleared supernatants from about 5×10^9^ cells were analysed by 15 % SDS-PAGE, followed by transfer onto nitrocellulose membrane and reaction with semi-purified anti-ParA antibody (Lasocki *et al.*, 2007). (b) and (c) Western blot analysis of ParB and ParA, respectively, in *parB1–18* mutant, *parB^+^* revertant (rev*B*^+^) and WT PAO1161 strains. Cells were collected at three stages of culture growth (indicated by OD_600_). Cleared extracts from about 10^9^ cells were analysed by 15 % SDS-PAGE followed by transfer onto nitrocellulose and immunodetection with Anti-ParB (b) and Anti-ParA (c) antibodies.

Since ParB and ParA interact with each other (Bartosik *et al.*, 2004) and in the light of the observation that ParB is highly unstable in *parA* null mutants (Lasocki *et al.*, 2007) it was important to determine whether any of the *parB* mutations affected the level of ParA. Samples of the *parB* mutants and WT as control were collected at a similar optical density and c.f.u. ml^−1^ were determined so that extracts from approximately 5×10^9^ cells could be separated by SDS-PAGE and analysed for the presence of ParA by Western blotting (Fig. 2a[Fig f2]). For *parB1–229*, *parB*Δ*121–183* and *parB1–90* the level of ParA was not significantly different from the level in WT cells (fluctuations may be due to the use of unsynchronized cultures) whereas it was undetectable in the extract prepared from the *parB* null mutant (*parB1–18*). Even a fivefold increase in the number of cells (2.5×10^10^) from the late exponential phase analysed on the gel gave no signal for ParA (Fig. 2a[Fig f2]; last track). As 50 ng ParA is visible under these conditions this means that the level of ParA in PAO1161*parB1–18* drops on average to below 40 molecules per cell. The *parB* mutant phenotypes described in the sections above were similar for all four analysed mutations so it is unlikely that the decrease in stability of ParA was the basis for any of the observed changes; ParA instability occurred in only one mutant, *parB1–18*.

### Localization of ParB in *P. aeruginosa* cells

**Fig. 3. f3:**
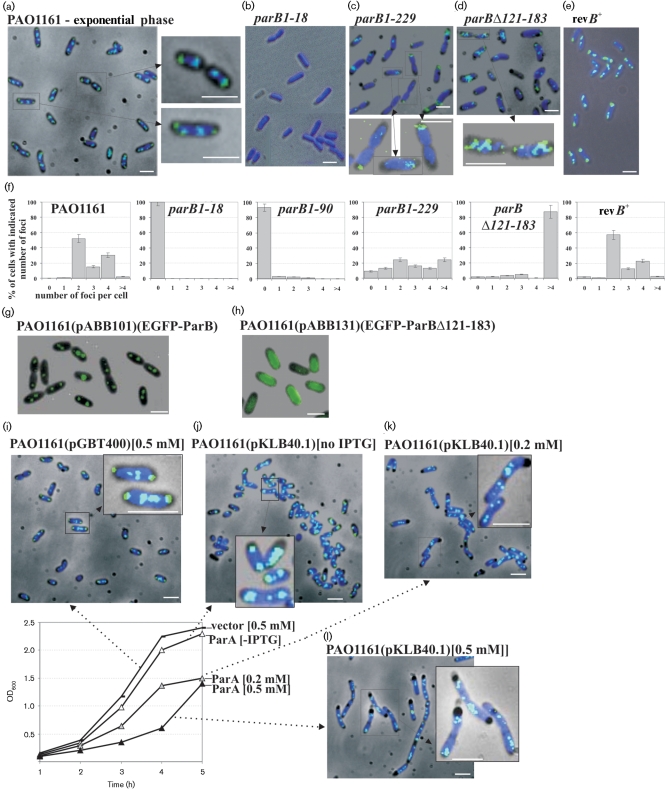
Cellular localization of ParB and its mutant forms in *P. aeruginosa*. (a–e) Localization of ParB in WT *P. aeruginosa*, mutant strains *parB1–18*, *parB1–229* and *parB*Δ*121–183*, and *parB*^+^ revertant strain (rev*B*^+^). Images show the location of ParB in the cells from the exponential phase of cultures grown in rich medium at 37 °C. Higher magnification of representative cells is also shown. Immunofluorescence and phase-contrast micrographs are overlaid. The dark background is a phase-contrast image, the dark blue is the DAPI-stained chromosome and the green/light blue is the FITC-stained ParB. (f) Bar charts showing the percentage of cells with different numbers of ParB foci for various strains. At least 500 cells were counted for each strain. (g, h) Overlaid phase-contrast and EGFP fluorescence images showing the cellular localization of the EGFP-ParB and EGFP-ParBΔ121–183 fusion proteins in living cells of *P. aeruginosa*. Cells from the PAO1161(pABB101) and PAO1161(pABB131) cultures, grown in L broth at 24 °C, were collected after 2–3 h induction with 0.2 mM IPTG and immediately prepared for microscopic examination. The dark background is the phase-contrast image and the green colour represents EGFP fused to ParB and ParBΔ121–183, respectively. (i–l) Immunofluorescence–phase-contrast overlay micrographs showing the localization of WT ParB in *P. aeruginosa* PAO1161(pKLB40.1*tacp-parA*) overproducing ParA protein. The graph presents the growth curves of PAO1161(pGBT400) and PAO1161(pKLB40.1) cultures grown in rich medium at 37 °C, from which the samples were collected for immunofluorescence microscopy. Different concentrations of IPTG were applied to overproduce ParA. PAO1161(pGBT400) cells grown in the presence of 0.5 mM IPTG were used as a control. The dark background is the phase-contrast image, the dark blue is the DAPI-stained chromosome and the green/light blue is the FITC-stained ParB. Scale bars 2 μm (all panels).

To determine the effect of the mutations on ParB localization within bacterial cells, two types of fluorescence microscopy were applied. Immunofluorescence microscopy with antibodies to ParB was applied to bacteria from both exponential- and stationary-phase cultures grown in rich medium at 37 °C. In the WT, the signal for DAPI-stained chromosomal DNA was found almost throughout the entire volume of the bacterial cell (Fig. 3a[Fig f3]). ParB formed specific foci apparently connected with the nucleoids. The majority of the cells from actively dividing cultures contained two to four ParB foci distributed symmetrically along the cell length (positions ^1^/_4_ and ^3^/_4_ or ^1^/_8_, ^3^/_8_, ^5^/_8_ and ^7^/_8_, respectively: Fig. 3a and f[Fig f3]). In a small percentage of cells (5–7 %) there were also multiple small foci of ParB next to the bigger, more compact ParB foci, suggesting that the bigger foci may be formed by condensation of smaller foci.

In the stationary-phase cultures the majority of cells had one or two foci of ParB (Supplementary Fig. S2B) and the FITC signals were much weaker in comparison to cells in exponential phase. In late stationary phase an extremely weak or no signal for ParB was detected in most of the cells. As the cells from stationary-phase culture may be more resistant to the fixing conditions a much harsher lysis method was used but the intensity of the ParB signal did not increase (data not shown). The level of ParB in stationary-phase cells of PAO1161 is about fivefold lower in comparison to the exponential phase (M. Andres & G. Jagura-Burdzy, unpublished results), which may explain the weaker signals for ParB in the cells from stationary-phase cultures.

For the *parB1–18* mutant strain the signal for ParB was not detected at all, as expected (Fig. 3b and f[Fig f3]). In the *parB1–90* mutant strain a very weak signal for ParB was observed only in less than 10 % of the cells and it did not exhibit a characteristic pattern of distribution (Fig. 3f[Fig f3]). The signal for ParB in two *parB* mutants, *parB1–229* and *parB*Δ*121–183*, was weaker than in WT bacteria (Fig. 3c and d[Fig f3]). The fluorescent signal of ParB1–229 was more dispersed and in the majority cells it was localized closer to the cell envelope (Fig. 3c and f[Fig f3]); ParBΔ121–183 formed multiple foci scattered irregularly in the cells (Fig. 3d and f[Fig f3]).

To follow changes in ParB distribution in living *P. aeruginosa* cells the EGFP protein (a more stable, red-shifted variant of WT green fluorescent protein from Clontech which has been optimized for brighter fluorescence) was fused to the N terminus of WT ParB and also to ParBΔ121–183. To confirm that the hybrid proteins were stable and functionally active they were tested for the previously observed ability of ParB*_P.a._*to silence genes that are linked to *parS* (Bartosik *et al.*, 2004). The observable effect of *repA* gene silencing in pGB2-*parS_2/3I_* (pABB811) caused by ParB is loss of the reporter plasmid. *E. coli* DH5*α*(pABB811) cells were transformed with pGBT30, pKLB2 and pABB301, so that the stability of pABB811 could be checked in the presence of the vector, WT ParB and EGFP-ParB, respectively. Strong destabilization of pABB811 was observed in the presence of excess ParB or EGFP-ParB (Supplementary Fig. S3A). This indicated that the fusion protein EGFP-ParB was functional in DNA binding and silencing. Overproduction of EGFP-ParBΔ121–183 (pABB331), lacking the DNA-binding domain, did not cause destabilization of the reporter plasmid, as was observed previously for ParBΔ121–183 (Bartosik *et al.*, 2004). To study the subcellular localization of ParB in *P. aeruginosa* cells, the fusions were transferred to the expression vector pGBT400, which is able to propagate in *P. aeruginosa*. Stability of the hybrid proteins in *P. aeruginosa* was confirmed by Western blotting (Supplementary Fig. S3B).

Fluorescence–phase-contrast combination microscopy of cells from exponential-phase cultures of PAO1161(pABB101 *lacI^Q^ tacp-egfp-parB*) showed that EGFP-ParB formed one to four foci, symmetrically distributed in living cells of *P. aeruginosa* (Fig. 3g[Fig f3]). These results corresponded to those of immunofluorescence microscopy on fixed cells. The EGFP-ParBΔ121–183 fusion protein in PAO1161(pABB131 *lacI^Q^ tacp-egfp-parBΔ121–183*) was unable to form foci, giving a dispersed signal throughout the cell (Fig. 3h[Fig f3]), as observed for EGFP on its own (data not shown).

The cells immobilized on the slides stayed alive for a long time so it was possible to observe changes in EGFP-ParB localization using time-lapse microscopy. Fluorescence–phase-contrast micrographs from the same field were collected at 2 min intervals. The experiment presented in Supplementary Fig. S2(C) showed that ParB foci undergo dynamic and rapid changes in approximately one-third of the cells. New foci of ParB were formed by duplication/splitting of pre-existing foci followed by rapid moving apart to give rise to regularly distributed foci in longer cells (four foci in mother cells before the completion of cell division).

### ParA overproduction affects formation of ParB foci and their distribution in *P. aeruginosa* cells

The overproduction of ParA*_P.a_*_._ is toxic for *P. aeruginosa* (Lasocki *et al.*, 2007). To see how an excess of ParA influences formation of ParB foci, PAO1161(pKLB40.1 =pGBT400*tacp-parA*) was grown in the absence and presence of IPTG, and cells were then collected and analysed by immunofluorescence microscopy. PAO1161(pGBT400) grown in the presence of 0.5 mM IPTG was used as a control and showed that neither the presence of the vector nor IPTG changed the distribution of ParB foci (Fig. 3i[Fig f3]). In fast-dividing cells, chromosomes initiate subsequent rounds of replication before finishing the previous cycle and it is assumed that there are at least two almost fully replicated genomes per cell. In the cells of PAO1161(pKLB40.1) grown without IPTG the ParB distribution was indistinguishable from the control (Fig. 3j[Fig f3]). Two or four ParB foci were distributed evenly within shorter and longer cells, respectively.

Addition of 0.2 mM or 0.5 mM IPTG to PAO1161(pKLB40.1) significantly reduced the growth rate (graph in Fig. 3) while cells appeared to be at least double the length of the control cells collected at the same growth phase (Fig. 3k and l[Fig f3]) with frequently occurring filamentous cells, some of them very long. Occasionally the nucleoids did not fill the whole cell, especially in the long filaments, leaving ‘empty’ spaces at the poles. In the elongated cells ParB did form foci but they were of much less regular pattern. The shortest observed cells exhibited ‘normal’ ParB foci but accompanied by a large number of smaller ParB foci, spread throughout the cell as the condensation of ParB foci was disturbed. In the fraction of the very long cells the larger ParB foci were evenly distributed along the filaments, but in the majority of the cells (more than 80 %) numerous ParB foci differing in size and number were observed that did not conform to the WT pattern of distribution. Some of the small ParB foci appeared to be preferentially positioned at the cell poles whereas in other cells they were localized in the middle of the cell (Fig. 3l[Fig f3]).

### Replacement of the mutated *parB1–18* : : *Tc^R^* allele by WT *parB* sequence restores the WT phenotypes

Introduction of mutations into *parB* might have generated secondary mutations in the genome, but since all four independent *parB* mutant strains had similar phenotypes (with the exception of the ParA level in the cells of the *parB1–18* mutant) this seemed unlikely. However, if we could complement the defects by providing *parB* from a different location then this would clearly demonstrate that the defect was the direct result of the introduced mutation.

To check if the observed phenotypes caused by mutations in *parB* could be complemented by ParB protein delivered *in trans* the WT *parB* with its ribosome-binding site was cloned under control of *tacp* in a medium-copy-number vector able to propagate in *P. aeruginosa*. None of the defective *parB* alleles could be complemented by the presence of *parB* delivered *in trans* (data not shown). Complementation tests for *parB* mutants were also carried out with both *parA* and *parB* genes delivered *in trans* but without success (data not shown). Western blot analysis showed that the amount of Par proteins delivered *in trans* (even without inducer) in a selection of transformants was about twofold higher than the level of Par proteins in the PAO1161 (data not shown).

Our preliminary data indicated that *parAparB* genes can be expressed at low level from a promoter internal to *gidB*. The region encompassing *gidBparAparB* was cloned into the suicide vector (pABB611) and forced to integrate into PAO1161*parB1–18* using homologous recombination. The levels of both ParB and ParA were found to be comparable with WT (data not shown); however, the integrants did not demonstrate the WT phenotypes. They grew slightly better than the *parB1–18* strain but still produced anucleate cells and were impaired in swarming and swimming motilities.

Finally it was decided to exchange the mutated allele *parB1–18* for the WT *parB* using the allelic exchange method on the integrants of PAO1161*parB1–18* : :pABB611 (El-Sayed *et al.*, 2001) and in this way to confirm the role of the *parB* mutation in the observed phenotypes. The production of full-length ParB at a level comparable to WT PAO1161 in the *parB^+^* revertant strain was demonstrated by Western blotting (Fig. 2b[Fig f2]). The growth rates of the tested *parB^+^* revertants were the same as that of PAO1161 (Fig. 1c[Fig f1]). The swarming and swimming motilities were also restored to normal in *parB^+^* strain (Fig. 1e[Fig f1]). The ParA content in cellular extracts of the revertant, verified by immunodetection, returned to the WT level (Fig. 2c[Fig f2]). Microscopic observations did not detect anucleate cells in the *parB^+^* revertant strain when approximately 5000 cells were examined (less than 0.02 %) (data not shown). Moreover the characteristic pattern of ParB localization was demonstrated by immunofluorescence microscopy (Fig. 3e and f[Fig f3]). These results confirmed that the pleiotropic defects observed in the *parB* mutant were the direct result of the *parB* allele and were not due to secondary mutations generated elsewhere in the chromosome.

## DISCUSSION

The primary focus of this study was to test the hypothesis that some or all of the phenotypes of a complete *parA* deletion mutant of *P. aeruginosa* could be due to destabilization of ParB. Because of the previous finding that loss of ParA results in disappearance of ParB (Lasocki *et al.*, 2007), we deliberately made both complete and partial knockouts of *parB*. The *in vitro* properties of the deletion mutant proteins, prior to studies *in vivo*, confirmed the growing body of data on this family of proteins. Thus removal of the C-terminal domain affected dimerization and reduced the affinity but not the specificity for *parS* sequences, while deletion of amino acids 121–183, which encompass the predicted HTH motif from amino acids 152 to 171, did not affect dimerization but did abolish DNA binding. When ParA and ParB levels were checked in the four constructed *parB* mutants it was discovered that ParA stability depends on ParB, suggesting that interaction between these two proteins forms a complex that stabilizes both proteins. Comparison of the *parB* mutants indicated that the N-terminal 90 aa is necessary for this stabilization. Thus the mutants of ParB that retain this region and therefore still produce normal ParA levels, but inactivate ParB function, enabled us to test our hypothesis.

ParA–ParB interactions seem to be a general rule for Par systems but the location of the ParB domain responsible for these interactions appears to vary in different systems (Autret *et al.*, 2001; Figge *et al.*, 2003; Kim & Shim, 1999; Lukaszewicz *et al.*, 2002; Ravin *et al.*, 2003; Surtees & Funnell, 1999). The finding that *P. aeruginosa* ParB1–90 was sufficient to preserve the normal level of ParA suggests that the N-terminal region of ParB interacts with ParA. This would be consistent with the observation that the ATPase activity of Soj (ParA) from *Thermus thermophilus* is stimulated by the presence of the first 20 aa of Spo0J (ParB) (Leonard *et al.*, 2005). This contrasts with our previous studies, where the C-terminal 56 aa of ParB was shown to be responsible for interactions with ParA (Bartosik *et al.*, 2004). It is possible that there are two separate domains within ParB that are important in ParB–ParA interactions – one in the N-tip and the other in the C-tip – although the removal of ParB sequence from the C terminus up to the N-terminal 90 aa did not reveal the second interaction domain with ParA in yeast two-hybrid studies (Bartosik *et al.*, 2004). Future detailed analysis will be necessary to resolve this, but for the purposes of this study the discrepancy is not important.

The ability to introduce *parB* mutations into the chromosome of *P. aeruginosa* indicates that this species, like the majority of other bacteria studied to date, e.g. *P. putida* (Godfrin-Estevenon *et al.*, 2002; Lewis *et al.*, 2002), *B. subtilis* (Ireton *et al.*, 1994; Lee *et al.*, 2003) and *Streptomyces coelicolor* (Kim *et al.*, 2000), remains viable without a functional *parB* gene, the exception being *C. crescentus*, where a lethal effect is observed (Mohl & Gober, 1997).

The *parB* mutants of *P. aeruginosa* grew more slowly than the WT (5–10 % longer division time) and the cells tended to be longer than WT. As only three out of four had insertion of the Tc^R^ cassette we can exclude the trivial explanation that the presence of the cassette induces the observed changes. All four *parB* mutants were impaired in nucleoid segregation, with more than 100-fold increase in the frequency of anucleate cells appearing (from <0.02 % for WT to 2–4 % in rich medium and 7 % in minimal medium), with many more cells demonstrating a guillotining effect during cell division without nucleoid separation. The perturbation in nucleoid separation was clearly visible in the exponential phase of growth, in contrast to the defect in *P. putida*, which was only seen during the transition into stationary phase (Godfrin-Estevenon *et al.*, 2002; Lewis *et al.*, 2002).

**Fig. 4. f4:**
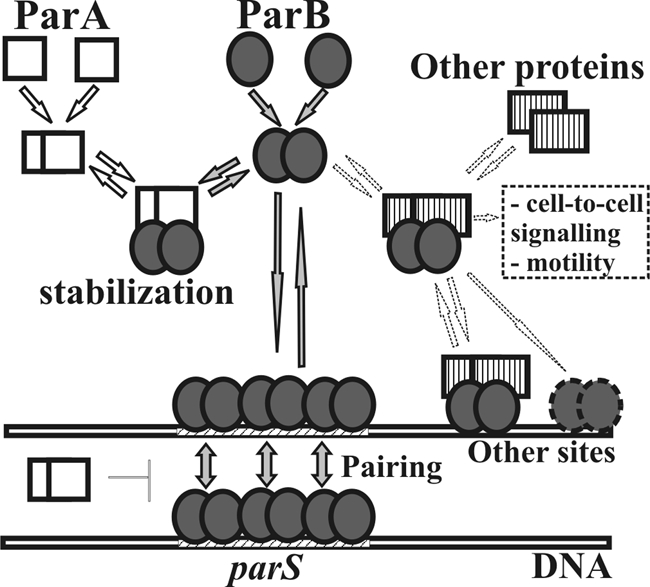
Model of action of Par proteins in *P. aeruginosa*. ParA and ParB proteins are able to interact with each other. ParB binds to *parS* sequences within the chromosome and spreads along DNA starting from *parS*. ParA may control the pairing of ParB–DNA complexes. ParB may also recognize other binding sites through interactions with other partners. Different complexes are involved in different cell processes such as chromosome segregation, cell division and motility.

The *parB* mutants were also drastically impaired in swarming and slightly in swimming activity, and formed irregular colonies with lamellar, jagged edges in contrast to the circular, regular colonies of the WT *P. aeruginosa* strain. The defects in motility may be the result of changes in cell-to-cell communication or flagellar structure/function, as flagellar function determines swarming and swimming abilities (Kohler *et al.*, 2000). Microscopic observation of PAO1161*parB1–18* cells showed the presence of flagella attached to one pole of the cell (data not shown) although at this stage the impairment of their rotary function cannot be excluded. Flagellar biogenesis is precisely controlled, and genes whose products participate in this process are clustered in different regions of the chromosome (PAO1 NC_002516; Dasgupta *et al.*, 2003). One of the predicted *parS* sites is adjacent to *pilGHIJK–chpABCD* whereas others are located 10–15 kb from *flgM flgN* (*parS_8_*), *motA motB* (*parS_9_*), *algZ algR* (*parS_10_*), operons involved in flagellar biogenesis, quorum-sensing regulation, signal transduction and chemotaxis necessary for twitching and swarming motilities, cell adherence and colonization (Darzins, 1994; Kohler *et al.*, 2000). Since ParB*_P.a._* is a DNA-binding protein and its interactions with particular *parS* sites can cause silencing of neighbouring genes if overproduced (Bartosik *et al.*, 2004), it is feasible that the observed defects in *P. aeruginosa parB* mutants are due to the overexpression of some loci when ParB is depleted. Spo0J, the ParB homologue of *B. subtilis*, was shown to spread laterally along DNA from *parS* nucleation sites covering more than 8 kb from particular *parS* sites (Murray *et al.*, 2006) although the latest studies did not confirm the significance of spreading in gene expression (Breier & Grossman, 2007). It is also likely that ParB may directly interact with the proteins involved in flagellar biogenesis or cell-to-cell signalling (model in Fig. 4); this hypothesis is currently being investigated.

Two visualization methods (immunofluorescence and EGFP) demonstrated that ParB in WT cells of *P. aeruginosa* forms regularly distributed foci (two in shorter cells and four in longer cells) located at the edges of the nucleoid. EGFP-ParB*_P.a_* fusion proteins in living cells showed that the new foci of ParB are formed by duplication of pre-existing foci and these quickly move apart (Supplementary Fig. S2C).

The ParB mutants defective in either the DNA-binding domain (but able to dimerize) or the C terminus, responsible for dimerization (still able to bind DNA but with lower specificity), lost the ability to form condensed specifically localized foci. ParBΔ121–183 formed several foci, random in number, whereas ParB1–229 was visible as tiny, multiple foci close to the cell poles, suggesting retention of some ability to interact and localize. Significantly, both of these ParB mutant forms possess an intact N terminus, suggested previously to be involved in interaction with cellular partners (Bartosik *et al.*, 2004). The recognition of cellular receptors may be required to promote the condensation and symmetrical distribution of ParB foci (Ebersbach *et al.*, 2008; Bowman *et al.*, 2008). The difference in ParBΔ121–183 signal depending on the visualization method used (immunofluorescence or EGFP) may be due to the presence of WT ParB. If there are receptors for ParB located in the cell membrane close to the poles they might be recognized by ParBΔ121–183 in the PAO1161*parB*Δ*121–183* mutant but have been preferentially occupied by the WT ParB in *P. aeruginosa* cells carrying both proteins (EGFP-ParBΔ121–183 and WT ParB), leading to the dispersion of the signal produced by EGFP-ParBΔ121–183.

Excess of ParA*_P.a._* leads to formation of elongated cells with abnormal ParB foci of different size and number. If the normal ratio of ParB to ParA allows ParB to spread from its binding sites and condense to form foci that can be separated by ParA, then excess ParA may interfere with this process because of a preponderance of ParA–ParB complexes and may also block other ParB interactions that are necessary for normal cellular functions.

The characterization of *parB* mutants of *P. aeruginosa* indicates the vital role of ParB in determining the functions impaired in both *parA* and *parB* mutants but it does not exclude the possibility that both ParA and ParB are involved in proper nucleoid segregation, growth rate and motility. It clearly demonstrates that ParA on its own is not sufficient to control those processes. Complementation studies of *par* mutants (Lasocki *et al.*, 2007 and this work) are very difficult as the small changes in the level of expression of *parA* and *parB* or slightly unbalanced production of both partners leads to the same phenotypes as observed for *parA* or *parB* mutants. We are aiming to isolate *parA* mutants producing ParB at WT level but unable to form an active ParA–ParB complex to discriminate between these possibilities.

On the basis of our current results we propose a working model for the action of Par proteins in *P. aeruginosa* cells (Fig. 4). In the cell ParA and ParB are expressed at a balanced level. Both proteins are able to form dimers and as such interact with each other (Bartosik *et al.*, 2004; K. Lasocki & G. Jagura-Burdzy unpublished). These interactions play an important stabilizing role for both proteins, and in the case of ParB the N-terminal 90 aa is vital for protection of ParA against degradation. ParB binds to *parS* sequences within the *P. aeruginosa* chromosome and is able to spread along DNA starting from *parS*. Our *in vivo* silencing studies in *E. coli* suggest that ParA is able to modulate ParB interactions with *parS* and formation of ParB–DNA complexes (A. Bartosik & G. Jagura-Burdzy, unpublished data), and these may be directly involved in nucleoid segregation. The model also predicts that ParB may interact with other cellular partners, e.g. the proteins involved in flagellar biogenesis or cell-to-cell signalling, and in complex with them or on its own may interact with sequences other than *parS* within the genome. Identifying such partners if they exist and defining the protein–protein interactions involved in this network should provide a much better understanding of the role of ParB in the cell biology of its host and will be the subject of future work.

## Figures and Tables

**Table 1. t1:** Plasmids used in this work

**Plasmid**	**Relevant features**	**Reference/source**
pABB211	pET28mod derivative with T7*p-parB1–229* fusion	Bartosik *et al.* (2004)
pABB330	pGBT30 derivative with *tacp-parB*Δ*121–183* fusion	Bartosik *et al.* (2004)
pABB811	pGB2 derivative with *parS_2/3I_* of *P. aeruginosa*	Bartosik *et al.* (2004)
pAKE600	*ori*_MB1_*ori*T_RK2_, Ap^R^*sacB*	El-Sayed *et al.* (2001)
pBGS18	*ori*_MB1_ Km^R^	Spratt *et al.* (1986)
pET28mod	*ori*_MB1_ Km^R^ T7p *lacO* His_6_ tag, modified to remove T7 tag	Lukaszewicz *et al.* (2002)
pGBT30	*ori*_MB1_ Ap^R^*lacI*^Q^*tacp*, expression vector	Jagura-Burdzy *et al.* (1991)
pGBT400	*ori*_RSF1010_ BHR Sm^R^*lacI*^Q^*tacp*, expression vector	Jagura-Burdzy *et al.* (1999)
pGEM-T Easy-*egfp*	pGEM-T Easy derivative with *egfp* cassette	Wei Zeng, PhD thesis, University of Birmingham
pKLB181	pUC18 with *parS_2/3_* on the *Hin*dIII–*Sal*I fragment	Bartosik *et al.* (2004)
pKLB2	pGBT30 with *tacp*-*parB* transcriptional fusion	Bartosik *et al.* (2004)
pKLB2.4	pGAD424 with *gal4_AD_-parB* translational fusion	Bartosik *et al.* (2004)
pKLB28	pET28mod derivative with T7*p-parB* fusion	Bartosik *et al.* (2004)
pKLB3	pGBT30 with *tacp*-_rbs_-*parAB* transcriptional fusion	Bartosik *et al.* (2004)
pKLB40.1	pGBT400 with *tacp*-*parA* transcriptional fusion	Lasocki *et al.* (2007)
pKLB60.1	pAKE600 without *Bam*HI site	Lasocki *et al.* (2007)
pKRP12	*ori*_MB1_ Ap^R^ Tc^R^ cassette	Reece & Phillips (1995)
pUC18	*ori*_MB1_ Ap^R^	Yanisch-Perron *et al.* (1985)
**Plasmids constructed during this study**		
pABB10	pUC18 with the *Eco*RI–*Sal*I fragment carrying *parB*	
pABB101	pGBT400 with the *Bam*HI–*Sal*I fragment of pABB301 carrying *lacI^Q^ tacp-egfp-parB*	
pABB131	pGBT400 with the *Bam*HI–*Sal*I fragment of pABB331 carrying *lacI^Q^ tacp-egfp-parB*Δ*121–183*	
pABB14	pUC18 with the *Eco*RI–*Sal*I fragment carrying the *parB1–90* : : *Tc^R^* disruption mutation	
pABB18	pBGS18 with the *Eco*RI–*Sal*I fragment carrying *parAparB1–18* : : *Tc^R^* from pABB318	
pABB230	pET28mod with the *Eco*RI–*Sal*I fragment carrying *parB*Δ*121–183*	
pABB24	pET28mod with the *Eco*RI–*Sal*I fragment carrying *parB1–90* : : *Tc^R^* from pABB14	
pABB301	pKLB2 with *Eco*RI fragment of pGEM-T Easy-*egfp* to form *egfp-parB* translational fusion	
pABB318	pGBT30 with *parAparB1–18* : : *Tc^R^*	
pABB331	pABB330 with *Eco*RI fragment of pGEM-T Easy-*egfp* to form *egfp-parB*Δ*121–183* translational fusion under *tacp* control	
pABB611	pAKE600 with the *Hin*dIII–*Sal*I fragment carrying *gidBparAparB*	
pABB614	pAKE600 with the *Eco*RI–*Sal*I fragment carrying *parB1–90* : : *Tc^R^* from pABB24	
pABB618	pAKE600 with the *Eco*RI–*Sal*I fragment carrying *parAparB1–18* : : *Tc^R^* from pABB18	
pABB660	pKLB60.1 with the *Eco*RI–*Hin*dIII fragment carrying *parB1–229*	
pABB661	pABB660 with the *Bam*HI–*Sal*I fragment carrying *parB235–290*	
pABB662	pABB661 with the Tc^R^ cassette inserted into the *Bam*HI site	
pABB663	pAKE600 with the *Eco*RI–*Sal*I fragment carrying *parB*Δ*121–183*	

## Abbreviations

- DAPI, 4′,6-diamidino-2-phenylindole
